# The Oncotype DX Recurrence Score's Impact on the Management of Oestrogen-Positive/Human Epidermal Growth Factor Receptor 2-Negative, Low-Burden Axillary Status Breast Cancer (REHAB Study): Results of a Single Centre

**DOI:** 10.7759/cureus.27341

**Published:** 2022-07-27

**Authors:** Abdalla Saad Abdalla Al-Zawi, Su-Lei Yin, Bayan Mahmood, Awais Jalil, Zina Aladili

**Affiliations:** 1 General and Breast Surgery, Mid and South Essex University Hospitals Group, Basildon, GBR; 2 General and Breast Surgery, Basildon and Thurrock University Hospital, Basildon, GBR; 3 General and Breast Surgery, Anglia Ruskin University, Chelmsford, GBR; 4 Oncology, Southend University Hospital, Southend-on-Sea, GBR

**Keywords:** axillary lymph node metastasis, human epidermal growth factor receptor-2, oestrogen receptors, chemotherapy, oncotype dx recurrence score, breast cancer

## Abstract

Background

The Oncotype DX Recurrence Score (ODX-RS) is increasingly utilized in oestrogen receptor (ER)-positive/human epidermal growth factor receptor 2 (HER2)-negative, low-burden axillary disease early operable breast cancer. It has been demonstrated to predict the benefits of adjuvant chemotherapy, hence supporting individualized decisions on adjuvant therapy.

Aim

To investigate the application of ODX-RS as an adjuvant treatment decision tool in breast cancer operated in our unit.

Methods

A total of 107 eligible patients who were operated on between 2017 and 2021 in Basildon University Hospital, UK were enrolled in this study. In this retrospective study, the clinical data, including patient’s age, tumour size, ER status, HER2 status, Ki67 proliferative index (Ki67-PI), nodal status, tumour grade, and ODX-RS, were collected. In the study design, the oncologist had the opportunity to assess the need for adjuvant chemotherapy for patients with ER-positive, HER2-negative, low-burden axillary lymph node disease, early breast cancer by using tumour characteristics and the PREDICT tool without knowing the ODX-RS results. The clinician's decision was matched against the breast multidisciplinary team's recommendations after ODX-RS utilisation, and the results were explored.

Results

The median ODX-RS of cohort tumours was 18 in the age group > 50 years, with ODX-RS ≥ 26 found in 18% of the group (n = 12). In the age group ≤ 50 years, 17% (n = 7) had ODX-RS between 21 and 25 and only 7% (n = 3) had ODX-RS ≥ 26. Without using ODX-RS, only 16% of the patients had been offered adjuvant chemotherapy in addition to the hormonal manipulation therapy; however, after using ODX-RS, up to 33% of the cohort was suitable for adjuvant chemotherapy in addition to the hormonal manipulation therapy. The changes in the recommendations after ODX-RS utilisation have been noticed in 29% of the cohort.

Conclusion

This study revealed that ODX-RS supported decision-making regarding postoperative adjuvant chemotherapy, especially when other tumour biomarkers, such as tumour size, grading, or Ki-67, indicated lower risk criteria. Patients with a high ODX-RS were offered chemotherapy where appropriate and its use led to a 15% rate of initial decision change in adjuvant treatment decisions; this involved either recommending chemotherapy or its omission.

## Introduction

The Oncotype DX Recurrence Score (ODX-RS) has been considered a crucial element in the management pathway of oestrogen receptor (ER)-positive, human epidermal growth factor receptor 2 (HER2)-negative, lymph node-negative/micrometastasis/N1 (one to three nodes positive) early invasive breast cancer and aids the physician to predict which patients would attain minimal or no benefit from adjuvant chemotherapy and would be safely spared from its adverse effects, without compromising outcomes [1]. On the other hand, it informs the physician about the patients associated with a higher risk of disease recurrence, and these patients will benefit from adjuvant chemotherapy in addition to hormonal manipulation treatment [2].

## Materials and methods

In this study, 107 patients who were operated on between 2017 and 2021 in Basildon University Hospital, UK were enrolled. This is a retrospective study where the clinical data, including patient’s age, tumour size, ER status, HER2 status, Ki67 proliferative index (Ki67-PI), nodal status, tumour grade, and ODX-RS, were collected. In the study design, the oncology team had the opportunity to assess patients’ need for adjuvant chemotherapy in addition to hormonal manipulation treatment by using tumour biological characteristics and the PREDICT tool. For the study cohort, the pre-ODX-RS adjuvant treatment oncologist’s recommendation was matched against the breast multidisciplinary team's decision after using the ODX-RS results, and the data were analysed.

## Results

**Figure 1:**
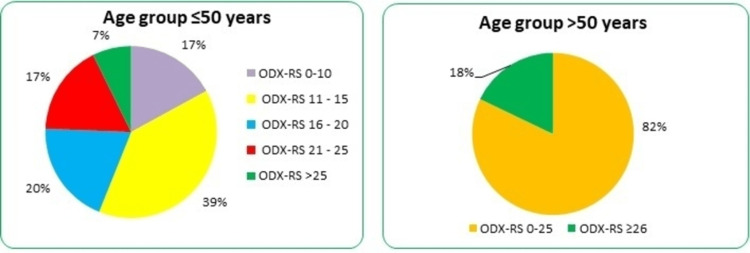
The ODX-RS results in the cohort according to the age groups. ODX-RS: Oncotype DX Recurrence Score.

Among the eligible 103 breast cancer patients, five had bilateral disease. The mean age was 55.8 ± 10.07 years. The total excised tumours were 108 lesions. Of them, 87% were T2 tumours, 8% were T1 tumours, and only 4.6% were T3 tumours. About 39% (n = 40) of the patients belonged to the age group ≤ 50 years, whereas 61% (n = 63) aged ˃ 50 years. The median ODX-RS of cohort tumours was 18 (range: 0-73). In the age group > 50 years, ODX-RS results were as follows: ≤25 (n = 55, 82%) and ≥26 (n = 12, 18%) (Figure 1). In the age group ≤ 50 years, 17% of patients (n = 7) had ODX-RS of <11, 39% (n = 16) had ODX-RS between 11 and 15, 20% (n = 8) had ODX-RS between 16 and 20, 17% (n = 7) had ODX-RS between 21 and 25, and only 7% (n = 3) had ODX-RS ≥ 26 (Figure 1).

In the cohort, 29 tumours (27.1%) showed borderline HER2 expression, approximately 55% of them were associated with lower Ki-67 (p = 0.63), and 93.1% (n = 27) of them had T2 tumour grade. Also, in the total cohort, the majority of the tumours (87%) were T2. Without knowing the ODX-RS results, the PREDICT tool was used to aid the physician in adjuvant chemotherapy decision-making. The results at this stage showed that only 16% of the patients could be offered adjuvant chemotherapy in addition to hormonal manipulation therapy. Those recommendations were matched against the multidisciplinary team's decisions after testing the ODX-RS.

**Figure 2:**
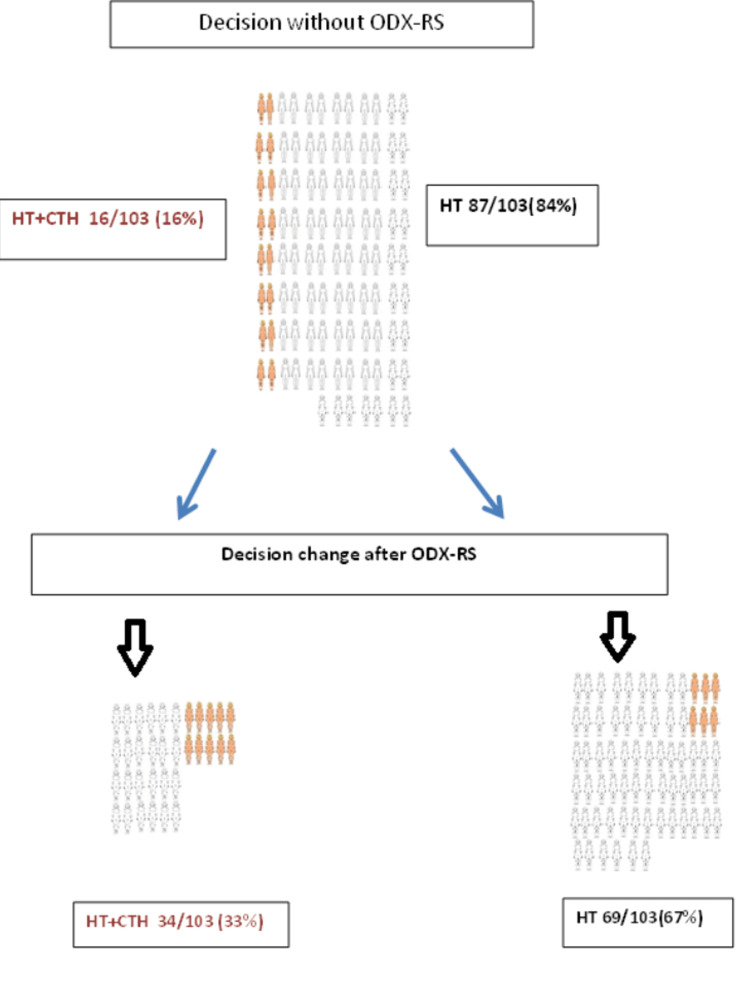
Adjuvant treatment recommendation change after ODX-RS utilisation. ODX-RS: Oncotype DX Recurrence Score; CTH: chemotherapy; HT: hormone therapy.

About 16% of the patients were recommended for hormone therapy + chemotherapy before ODX-RS utilisation, and 62% of them had their treatment changed to hormone therapy only after ODX-RS utilisation. On other hand, 84% (n = 87) of the cohort had recommendations for hormone therapy only before ODX-RS utilisation, and 28% of them (n = 24) had treatment changed to hormone therapy + chemotherapy after ODX-RS utilisation (Figure 2).

**Figure 3:**
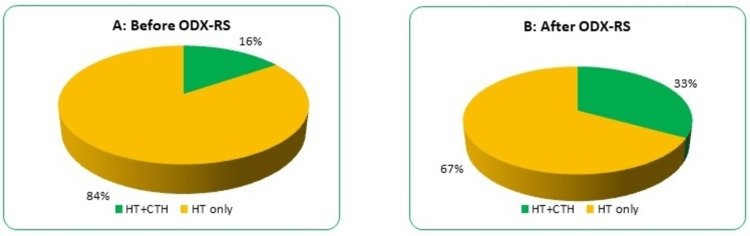
Impact of ODX-RS on breast cancer management. (A) Recommendation before ODX-RS. (B) Change in treatment recommendation after ODX-RS. ODX-RS: Oncotype DX Recurrence Score; CTH: chemotherapy; HT: hormone therapy.

The changes in the recommendations after ODX-RS utilisation have been noticed in 29% of the total cohort. Finally, in this study, up to 33% of the cohort was suitable for adjuvant chemotherapy in addition to hormonal manipulation (Figure 3).

**Figure 4:**
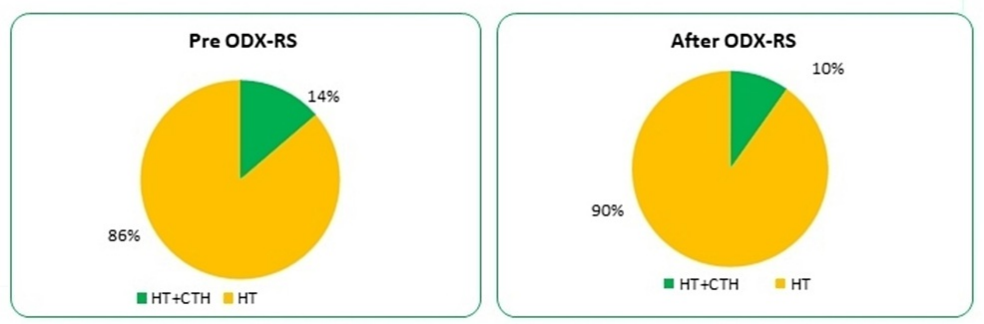
Pre and post-ODX-RS adjuvant treatment recommendations in patients with low Ki-67 proliferation index. ODX-RS: Oncotype DX Recurrence Score; CTH: chemotherapy; HT: hormone therapy.

The authors analysed the impact of Ki67-PI on such a situation, and the 10% value has been used as a cut-off point. The results of ≤10% were categorised as low expression and values of >10% were classified as high KI67-PI. In the group with low Ki67-PI expression, about 14% had chemotherapy recommended/offered without knowing ODX-RS results; this has been reduced to 10% only after using ODX-RS (Figure 4).

**Figure 5:**
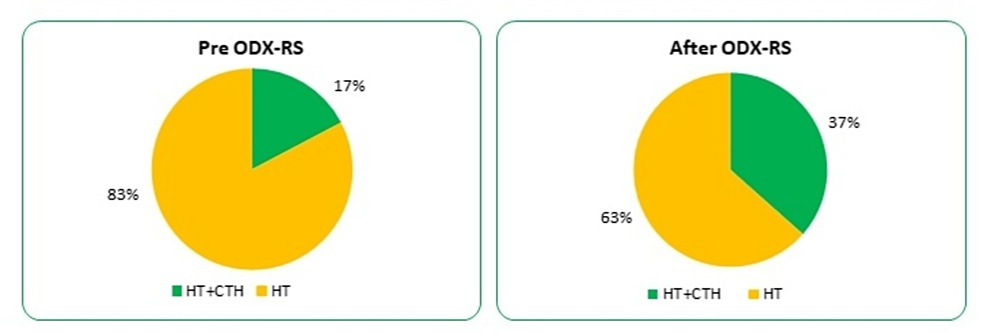
Pre and post-ODX-RS adjuvant treatment recommendations in patients with high Ki-67 proliferation index. ODX-RS: Oncotype DX Recurrence Score; CTH: chemotherapy; HT: hormone therapy.

In the category of high Ki67-PI, only 17% had chemotherapy recommended/offered without knowing ODX-RS results; this has changed to 37% after using ODX-RS (p = 0.89) (Figure 5).

About 10% of patients with low Ki67-PI showed a high ODX-RS; accordingly, chemotherapy had been recommended for them. Also, 8% of the low Ki67-PI group had chemotherapy indicated prior to ODX-RS, and this remained unchanged (ODX-RS ranged between 12 and 24).

## Discussion

**Figure 6:**
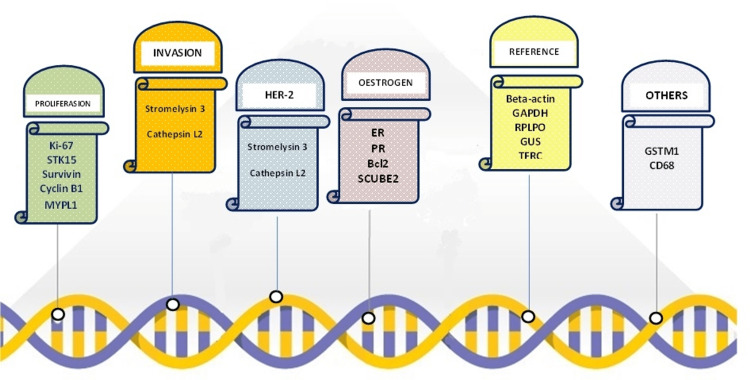
The ODX-RS assay 21-gene panel. Adapted from [3]. ODX-RS: Oncotype DX Recurrence Score.

Worldwide, breast cancer is the most frequent type of malignancy in females. Its incidence is climbing every year and this trend is expected to continue [3,4]. In the UK, it is recognised as the most common cancer with 55,000 new cases detected every year, and it is responsible for 7% of all cancer deaths [5]. ER-positive, HER2-negative, invasive breast cancer phenotype represents the most common subtype of breast cancer, accounting for 75% of all diagnosed invasive breast cancers [6]. Despite the fact that less than 10% of ER-positive, HER2-negative, low-burden axillary disease, early invasive breast cancers benefit from chemotherapy [7], the utilization of adjuvant chemotherapy in the management of ER-positive early breast cancer has resulted in the reduction of breast cancer-related mortality [8]. These patients may present with locoregional disease (breast with/without early axillary lymph node disease), which would usually suggest a good prognosis. Yet, some of them possess a notable risk of cancer recurrence after treatment [1]. The ODX-RS assay panel is composed of a total of 21 genes. The quantitative analysis of the gene expression involves five reference genes (ACTB, TFRC, GAPDH, GUSB, and RPLP0) and 16 cancer-related genes. The cancer genes are composed of genes that code for HER2 (HER2 and GRB7), oestrogen genes (ER, PGR, BCL2, and SCUBE2), proliferation genes (MKI67, STK15, BIRC5, CCNB1, and MYBL2), and invasion genes (MMP11 and CTSL2) as well as GSTM1, CD68, and BAG1 (Figure 6) [3].

**Figure 7:**
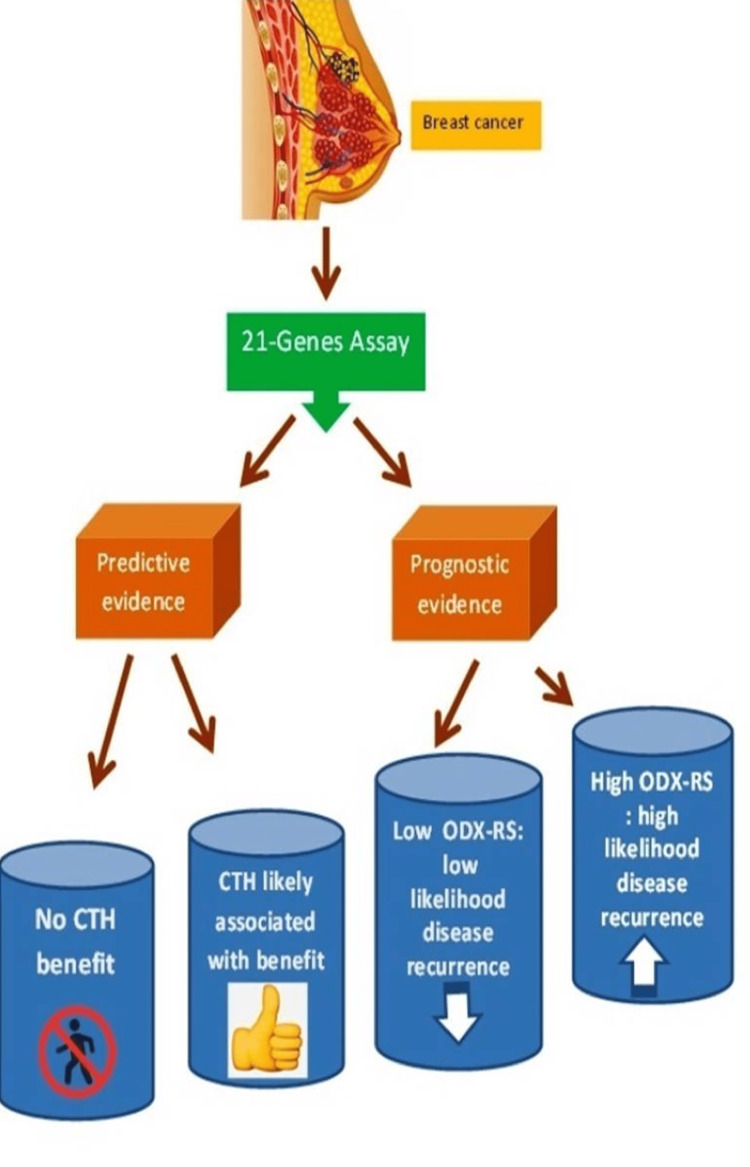
The role of ODX-RS in adjuvant chemotherapy decision-making for ER-positive, HER2-negative, low-burden axillary disease, early breast cancer. ODX-RS: Oncotype DX Recurrence Score; CTH: chemotherapy; ER: oestrogen receptor; HER2: human epidermal growth factor receptor 2.

The ODX-RS assay is of both predictive value for potential treatment benefit as well as prognostic merit and provides more information regarding possible management outcomes; broadly speaking, it leads the way towards better treatment individualisation (Figure 7).

The HER2-negative subtype of invasive breast cancer includes two genomically distinct categories, labelled as luminal A and luminal B. The latter group has a diminished response to hormonal manipulation therapy and is associated with an inferior prognosis [9]. Therefore, the concealed genetic features of the tumour may be more influential in predicting disease behaviour than the patient’s particular characteristics of the tumour size, grade, and stage at the time of disease presentation. Analysing the results of the Trial Assigning Individualized Options for Treatment-Rx (TAILORx) clinical trial low ODX-RS (<11) arm data from 2015, it was reported that hormonal manipulation treatment alone is associated with good long-term outcomes; this suggests that patients with low ODX-RS should generally be spared from chemotherapy [10,11].

Nishimura et al. (2010) presented a report related to the clinical significance of Ki67-PI in neoadjuvant chemotherapy for primary breast cancer. The authors reported that the pre-treatment Ki67-PI value prior to the upfront chemotherapy was a critical predictive factor for the effectiveness of neoadjuvant chemotherapy, and the Ki67-PI values after upfront chemotherapy completion were remarkably decreased and correlated with clinical response and disease-free survival (DFS) period; generally speaking, the lower the Ki67-PI value, the longer and favourable the DFS [12,13]. In our study, we have used the 10% value as the Ki67-PI cut-off point. The adjuvant chemotherapy indication has increased by more than 100% after ODX-RS results utilization in the high Ki67-PI group (37% vs. 17%) (Figure 5).

In the TAILORx clinical trial report from 2018, a total of 6711 patients with a midrange ODX-RS between 11 and 25 were enrolled and were randomly assigned to receive either chemo-endocrine therapy or endocrine therapy alone. The authors concluded that adjuvant hormonal manipulation therapy and chemo-endocrine therapy had similar efficacy in patients with hormone-receptor-positive, HER2-negative, axillary node-negative breast cancer who had a midrange ODX-RS, despite the fact that some benefit of chemotherapy was established in some women of ≤50 years of age [14]. As we see here, ODX-RS is not solely an indicator of disease prognosis; however, more importantly, it predicts the potential responsiveness for adjuvant chemotherapy.

Ki-67, also known as MKI67 (marker of proliferation Ki-67), is a protein found in actively dividing cells. It is detected in the nuclei of proliferating cells in the G1, S, G2, and M phases of the cell cycle. Its levels are low during the G1 and early S phase, and as the cell division progresses, Ki-67 concentration gradually increases to reach its maximum level during mitosis. For this reason, the Ki67-PI can be a useful marker of cell proliferation [13,15]. The published reports showed that Ki67-PI is related to the disease prognosis and the response to systemic therapy in both the adjuvant and neoadjuvant treatment settings. It is associated with an increased risk of disease recurrence in breast cancer as well as increased breast cancer-related mortality [16].

Walter et al. (2020) presented a retrospective analysis of 4695 patients with hormone receptor-positive and HER2-negative early breast cancer (T1-3: tumour size ≤ 5m; N0-1: node-negative/micrometastasis/one to three nodes positive; M0: no distant metastasis), who had been tested for ODX-RS in Germany in the period between November 2015 and July 2018. The authors were following the TAILORx trial for ODX-RS categorization: low (0-10), midrange (11-25), and high (26-100). The results revealed that 21% of patients were assigned to the low ODX-RS group, 63% to the midrange group, and 15% to the high score group.

The authors also explored the other tumour biomarkers in relation to ODX-RS and found that under the combination of low and midrange ODX-RS groups, there will be 81% of node-negative patients over 50 years of age, 90% of node-positive patients, 79% among tumours with Ki-67-high (≥20%), 86% of grade 2 tumours, 70% of grade 3 tumours, 88% of patients with T3 tumours, and 82% among node-negative patients with high-risk tumours. The paper concluded that chemotherapy may not be beneficial in most of the tested groups. Here, we have to highlight the importance of the benefit of the ODX-RS in sparing a good number of patients from the unnecessary adverse effects of chemotherapy [17].

Results of the REHAB study reveal that the utilization of ODX-RS assay has significantly impacted the chemotherapy indication and could lead to a change in adjuvant treatment recommendation in up to 29% of patients for whom the physicians, to begin with, were initially facing uncertainty regarding treatment option based on classical clinical and tumour biological markers. As shown in Table 1, our figures are similar to most of the previously published data [18-30].

**Table 1:** Change in treatment recommendations after ODX-RS utilisation (literature data).

Author (year)	Cohort size	Rate
Holt et al. (2013) [18]	142	26.8%
Eiermann et al. (2013) [19]	366	33%
Yamauchi et al. (2014) [20]	124	33%
Jaafar et al. (2014) [21]	47	27.7%
Gligorov et al. (2015) [22]	100	37%
Bargallo et al. (2015) [23]	96	32%
Albanell et al. (2016) [24]	527	32%
Kuchel et al. (2016) [25]	135	22.7%
Levine et al. (2016) [26]	979	38%
Leung et al. (2016) [27]	146	23%
Dieci et al. (2019) [28]	251	30%
Fayaz et al. (2020) [29]	100	37%
Mattar et al. (2021) [30]	179	30%

However, heterogeneity of the results and the differences in rates between the presented cohorts can be accounted for by sample size variation, cohorts' age range, menopausal status, test availability, and patient selection. Also, tumour biomarkers such as grade and size in addition to lymph node status and Ki67-PI are used as cut-off points. Some authors suggest that some factors such as tumour grade might serve as a guide during decision-making for patients with whom the ODX-RS test can be avoided and just recommend endocrine therapy alone in those low-risk patients; also, they suggest recommending the adjuvant chemotherapy for ER-positive, HER2-negative, N0 high-risk patients without testing the ODX-RS such as those having large, high-grade, or progesterone receptor-negative tumours [26]. Our study was limited, as are others, by the small sample size and lack of long-term patient follow-up in addition to the patient selection issue, especially after the testing for Nmic/N1 patients was introduced into practice.

## Conclusions

This study revealed that ODX-RS supported decision-making regarding postoperative adjuvant chemotherapy, especially when other tumour biomarkers, such as tumour size, grading, and Ki-67, indicated lower risk criteria. Patients with a high ODX-RS were offered chemotherapy where appropriate, and its use led to a 15% rate of change in the adjuvant treatment decision.
